# Efficacy of pillar suture for post-tonsillectomy morbidity in children: a meta-analysis

**DOI:** 10.1016/j.bjorl.2019.12.007

**Published:** 2020-01-25

**Authors:** Ji-Sun Kim, Byung Guk Kim, Dong-Hyun Kim, Se Hwan Hwang

**Affiliations:** The Catholic University of Korea, College of Medicine, Department of Otolaryngology-Head and Neck Surgery, Seoul, Republic of Korea

**Keywords:** Tonsillectomy, Pillar suture, Postoperative hemorrhage, Postoperative pain, Meta-analysis

## Abstract

**Introduction:**

Several surgical techniques have been used during tonsillectomy to reduce complications.

**Objectives:**

To assess the effects of pillar suture in conjunction with tonsillectomy as compared to tonsillectomy without suture in children.

**Methods:**

Two authors independently searched five databases (PubMed, SCOPUS, Embase, the Web of Science, and the Cochrane database) for studies published as recent as December 2018. Of the included studies, we compared tonsillectomy and pillar suture in combination (suture groups) with tonsillectomy alone,without suture, (control group). Postoperative pain intensity and other morbidities (e.g., postoperative bleeding, palatal hematoma, discomfort, and pillar edema) were measured during the postoperative period.

**Results:**

Postoperative bleeding [primary (OR = 0.47 [0.27; 0.81]) and secondary (OR = 0.14 [0.02; 0.78]) were significantly decreased in the pillar suture group compared to the control group. There were no significant differences between the two groups in postoperative pain at day 7 (SMD = −0.39 [−0.79; 0.00]), palatal hematoma (OR = 5.00 [0.22; 112.88]), palatal discomfort sensation (OR = 2.62 [0.60; 11.46]), site infection (OR = 5.27 [0.24; 113.35]), and velopharyngeal insufficiency (OR = 2.82 [0.11; 74.51]). By contrast, pillar edema (OR = 9.55 [4.29; 21.29]) was significantly increased in the pillar suture group compared to the control group.

**Conclusions:**

Pillar suture combined with tonsillectomy may reduce postoperative bleeding incidence despite increasing pillar edema in pediatric tonsillectomy. Postoperative pain-relief, palatal hematoma, palatal discomfort sensation, site infection, and velopharyngeal insufficiency were not significantly altered compared to tonsillectomy alone. However, further studies are needed to corroborate the results of this study.

## Introduction

Tonsillectomy is a common surgical procedure in otolaryngology, which is usually performed for frequent tonsillitis or sleep-disorder breathing. Typical postoperative complications include pain and bleeding. Several methods have been used during the surgical procedure in an attempt to reduce complications. These have included injecting drugs such as tramadol, morphine, and steroids into the peritonsillar area, applying fibrin glue to the surgical site, and obliterating the tonsillar fossa by manipulating the pillars.^1^, ^2^, ^3^, ^4^, ^5^, ^6^

There have been studies reporting the effectiveness of pillar suture in conjunction with a tonsillectomy. The pillar suture is a method of suturing the anterior or posterior pillar on the exposed tonsillar fossa after tonsillectomy, or suturing the anterior and posterior pillars together. Suture techniques vary among surgeons. The goal of these techniques is to reduce the area of ​​exposed pharyngeal muscle after tonsil removal, thereby reducing irritation caused by postoperative swallowing and promoting rapid healing through epithelization of the surgical site.^7^ There have been reports that pillar suture can reduce postoperative pain and bleeding.^8^, ^9^, ^10^ However, the necessity of the pillar suture is debatable since there have been studies showing conflicting results.^11^, ^12^

It is important to have a comprehensive understanding of the various criteria and results that determine the effectiveness of pillar suture. Therefore, the purpose of this study is to review the effects of pillar suture in tonsillectomy. The effects of this technique were evaluated by postoperative pain, postoperative bleeding, pillar edema, palatal hematoma, palatal discomfort sensation, suture site infection, and velopharyngeal insufficiency.

## Methods

### Literature search strategy

Clinical studies published up to December 2018 were identified from PubMed, SCOPUS, Embase, the Web of Science, and the Cochrane Central Register of Controlled Trials. The search terms were as follows: ‘tonsillectomy’, ‘adenotonsillectomy’, ‘suture’, ‘pharyngoplasty’, ‘fossa obliteration’, ‘children’, ‘pain’, ‘bleeding’, ‘velopharyngeal insufficiency’, ‘edema’, ‘discomfort’, and ‘analgesics’. Only studies written in English were included. References of searched studies were identified to ensure that no related studies were overlooked.

Two reviewers, working independently, scanned all abstracts and titles for candidate studies and removed studies that were not associated with pillar suture during tonsillectomy.

### Selection criteria

**Figure 1:**
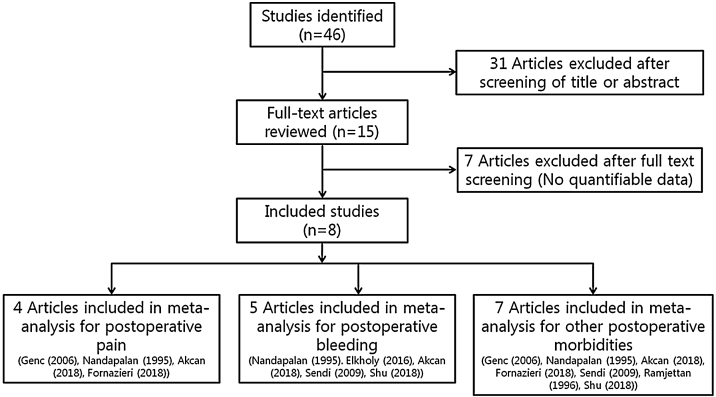
Diagram of the selection of studies for meta-analysis.

Randomized controlled trials that studied patients undergoing tonsillectomy procedures regardless of methods (cold knife or thermal dissection) were eligible for review. Studies were not adequate for inclusion if, in addition to adenotonsillectomy, patients had undergone other operations such as nasal and otologic surgery, or if reports were duplicated. In addition, studies were excluded from the analysis if clinical outcomes were not clearly described with quantifiable data, or if it was not possible to evaluate the data from the described results. The search strategy summarizes to screen studies chosen for meta-analysis (Fig. 1).

### Data extraction and risk of bias assessment

Data from studies were extracted with standardized forms and identified by two reviewers working separately. Outcomes for analysis included postoperative pain score on day 7, postoperative bleeding (incidence of primary or secondary bleeding), pillar edema (incidence of pillar edema), palatal hematoma (incidence of palatal hematoma), palatal discomfort sensation (incidence of palatal discomfort sensation), suture site infection (incidence of suture site infection), and velopharyngeal insufficiency (incidence of velopharyngeal insufficiency). We defined primary hemorrhage as bleeding within 24 h after surgery, and secondary hemorrhage as all later bleeding.^13^ The outcomes were compared between a group receiving both pillar suture and tonsillectomy, and a control group as defined by patients receiving conventional tonsillectomy without pillar suture.

The effects of pillar suture on postoperative morbidities were assessed based on discrete studies of patients following their departure from the operation room. From the included studies, we extracted the following data: the number of patients; the grading scale or score used to quantify pain; the incidence or percentage of postoperative bleeding, pillar edema, palatal hematoma, palatal discomfort sensation, suture site infection, and velopharyngeal insufficiency; and the p-value for the comparison between the pillar suture and control groups. The risk of bias for each study was analyzed with the Cochrane ‘Risk of Bias’ tool.

### Statistical analysis and outcome measurements

The ‘R’ statistical software (R Foundation for Statistical Computing, Vienna, Austria) was utilized for the meta-analysis of the selected studies. For continuous variables, meta-analysis was conducted with the Standardized Mean Difference (SMD). The individual mean difference (treatment outcome minus control outcome) was assigned a weight according to the size and standard deviation of each study in order to provide a precise estimate of treatment effect and then summated into a single outcome. For incidence related variables, Odds Ratio (OR) was utilized according to the Mantel-Haenszel method. Heterogeneity was evaluated with the I^2^ statistic.

## Results

A total of eight studies with 1712 participants were evaluated for the meta-analysis. The results of bias assessment and study characteristics are described in Table 1. Publication bias was not measured because the number of trials analyzed was insufficient to properly measure with a funnel plot or to perform more advanced regression-based assessments.

**Table 1:** Summary of studies included in the meta-analysis.

Leading author (year)/Operation type	Number of patients/Age range	Study type	Comparison (suture material/Coagulation)	Outcome measure analyzed	Judgment of risk of bias
Genc (2006) / Dissection and snare	39 (male 22: female 17/ 3–15)	Randomized controlled prospective study	One pillar suture side vs. the other control side (4/0 plain catgut sutures/compression and diathermy)	Pain scores (Wong-Baker faces scale) Postoperative morbidity (pillar edema, suture site infection)	High
Nandapalan (1995) / Dissection and snare	49 (not identified / 3–15)	Randomized controlled prospective study	One pillar suture side vs the other control side (2.0 Polydioxanone/ diathermy)	Pain scores (visual analogue scale) Postoperative morbidity (postoperative bleeding, palatal hematoma, palatal discomfort sensation)	High
Elkholy (2016) / Dissection	800 (male 404: female 396 / 3–20)	Randomized controlled prospective study	Pillar suture group vs. Control group (Chromic gut or vicryl/ compression, diathermy, or ligation)	Postoperative morbidity (postoperative bleeding, palatal hematoma, palatal discomfort sensation)	Unclear
Akcan (2018) / Bipolar diathermy	79 (male 55: female 24/ mean age 6.5)	Randomized controlled prospective study	Pillar suture group vs. Control group (4/0 vicryl/ diathermy)	Pain scores (Wong-Baker faces scale) Postoperative morbidity (postoperative bleeding, suture site infection)	Low
Fornazieri (2018) / Dissection	80 (male 41: female 39 / 5∼12)	Randomized controlled prospective study	Pillar suture group vs. Control group (Chromic gut/ diathermy)	Pain scores (Wong-Baker faces scale) Postoperative morbidity (pillar edema)	Low
Sendi (2009) / Dissection and snare	36 (male 16: female 20 / 7∼16)	Randomized controlled prospective study	One pillar suture side vs. the other control side (Chromic gut/ diathermy)	Postoperative morbidity (postoperative bleeding, suture site infection)	High
Ramjettan (1996) / Dissection and snare	32 (not identified)	Randomized controlled prospective study	Pillar suture group vs. Control group (Chromic gut /diathermy)	Postoperative morbidity (palatal hematoma)	Unclear
Shu (2018) / Dissection and snare	602 (male 245: female 357 / mean age 4.)	Randomized controlled prospective study	Pillar suture group vs. Control group (chromic gut/ diathermy)	Postoperative morbidity (postoperative bleeding, palatal discomfort sensation)	low

### Application of pillar suture compared to control (postoperative pain)

**Figure 2:**
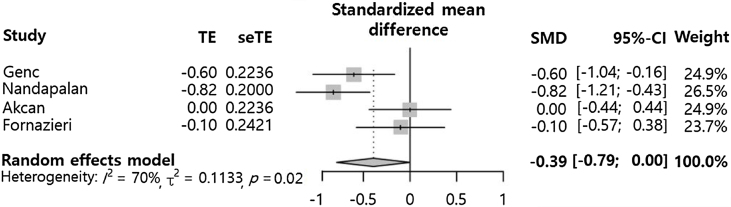
Pillar suture versus control groups for postoperative pain on postoperative day 7. (TE, Treatment Effect; seTE, Standard Error of Treatment Effect; SMD, Standardized Mean Difference; CI, Confidence Interval).

Postoperative pain at 7 days (SMD = −0.39 [−0.79; 0.00], p-value = 0.0529, I^2^ = 69.8%) tended to decrease in the pillar suture group compared to the control group (Fig. 2). However, there was no statistically significant difference between the groups. Significant inter-study heterogeneity was found for this outcome (I^2^ > 50%).

The overall analysis did not consider study design (i.e. paired samples comparing pillar suture side to the non-operated side or independent samples comparing the pillar suture group to a non-operated group). This could explain the high heterogeneity (more than 60 percent) seen in the results. Eventually, subgroup analyses were conducted. In cases of comparisons with single patients, pillar suture showed a significant pain-relieving effect (SMD = −0.72 [−1.01; −0.43], I^2^ = 0%). In contrast, comparisons between different patient groups showed this additional procedure had no significant beneficial effect on postoperative pain (SMD = −0.04 [−0.36; 0.27], I^2^ = 0%). Analysis of the effect of pillar suture according to study design showed that the difference in comparison methods potentially influenced outcomes.

### Application of pillar suture compared to control (other postoperative morbidities)

**Figure 3:**
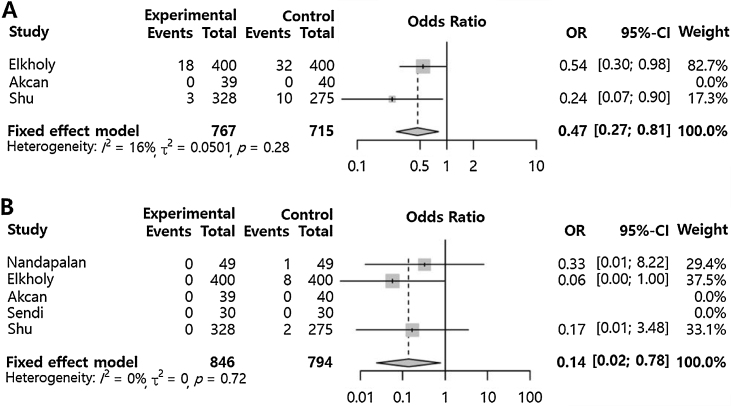
Pillar suture versus control groups for postoperative bleeding. Odds Ratio of the incidence of primary postoperative bleeding (A) and secondary postoperative bleeding (B). (Total, Nnumber of participants per group; OR, Odds Ratio; CI, Confidence Interval).

**Figure 4:**
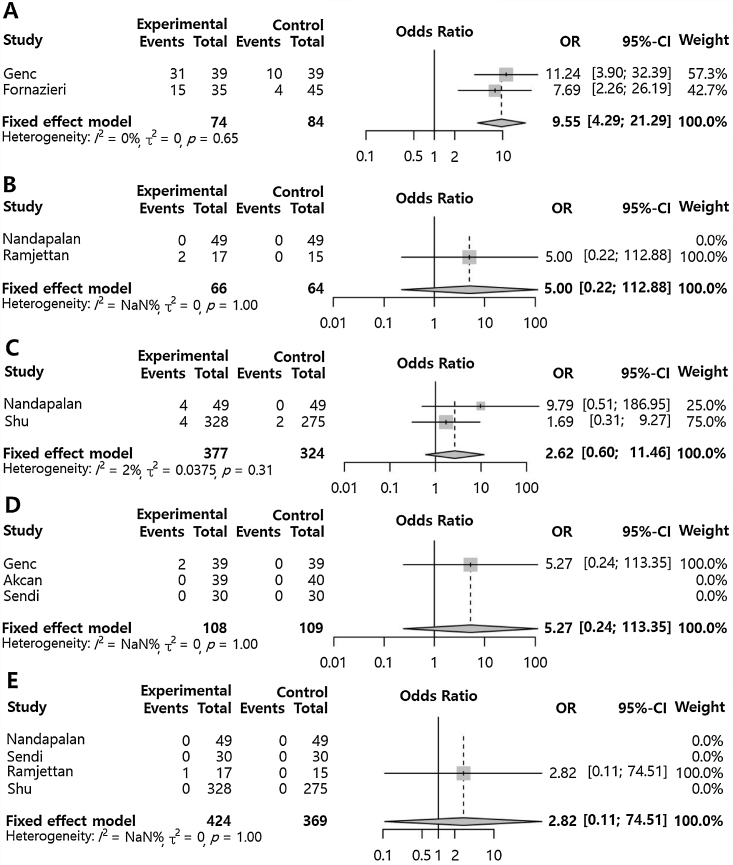
Pillar suture versus control groups for postoperative adverse effects. Odds ratio of the incidence of pillar edema (A), palatal hematoma (B), palatal discomfort sensation (C), suture site infection (D), and velopharyngeal insufficiency (E). (Total, Number of participants per group; OR, Odds Ratio; CI, Confidence Interval).

Primary postoperative bleeding (OR = 0.47 [0.27; 0.81], p-value = 0.006, I^2^ = 16%) and secondary postoperative bleeding (OR = 0.14 [0.02; 0.78], p-value = 0.025, I^2^ = 0%) were significantly lower in the pillar suture group compared to the control group (Fig. 3). However, pillar edema (OR = 9.55 [4.29; 21.29], p-value < 0.001, I^2^ = 0) was significantly increased in the pillar suture group compared to the control group (Fig. 4). There were no significant differences in palatal hematoma (OR = 5.00 [0.22; 112.88], p-value = 0.3115, I^2^ = NA), palatal discomfort sensation (OR = 2.62 [0.60; 11.46], p-value = 0.2012, I^2^ = 2.4%), suture site infection (OR = 5.27 [0.24; 113.35], p-value = 0.2887, I^2^ = NA), and velopharyngeal insufficiency (OR = 2.82 [0.11; 74.51], p-value = 0.5352, I^2^ = NA). No inter-study heterogeneity was found in postoperative morbidities compared to the control (I^2^ < 50%).

## Discussion

Postoperative pain may prolong the length of hospital stay, cause discomfort for the child and the caregiver, and affect the nervous, endocrine, and immune systems in a variety of ways.^14^, ^15^ The sensory nerve endings at the surgical site are exposed and stimulated by saliva and food, which is one of the causes of post-tonsillectomy pain.^5^ There have been several studies evaluating postoperative pain relief by covering exposed nerve endings on the open tonsillar fossa using various grafting materials.^16^, ^17^ The pillar suture could also have an effect on postoperative pain by covering exposed nerve endings.

Epithelial coverage in the tonsillectomy bed has been reported to develop gradually after postoperative day 7, which is when the fibrin clot is separated from the operative site.^18^ In our study, pillar suture tended to decrease postoperative pain at day 7 compared to the control group. Although not statistically significant, this trend could be explained by coverage of the open tonsillar fossa with pillar closure. In addition, the pillar suture group was found to have significantly faster wound healing than was found in the control group.^9^ This observation could also explain why the pillar suture reduces pain. However, contrary to our expectation for this intervention’s effects, our results showed a small and nonsignificant effect size. Pain after tonsillectomy has been reported to be more prominent in children older than 10 years.^19^ There has been a report that the pain relief effect derived from pillar suture is greater in children than in adults.^20^ These results could mean that postoperative pain and the pillar suture effect could be influenced by age. However, in our study, patient ages in the included studies ranged from 3 to 20 years. The wide age range could partially explain the non-significant but favorable pain effect of pillar suture. Further studies are needed to determine the effects of pillar suture according to child age.

In addition, the subgroup analyses of postoperative pain in this study showed different results according to study design. Pillar suture showed a significant reduction in postoperative pain only in subgroups comparing both sides in a single patient. The pain-relieving effect of pillar suture was not significant in subgroups that compared pillar suture patient groups with control groups. This discrepancy could explain the high heterogeneity and the lack of statistical significance in the summated outcome. Each study design has its advantages and disadvantages. For example, the design comparing pain on two sides in the same patient would have the advantage of including tonsillectomies conducted by the same surgeon and of having identical postoperative time in the same patient.^7^ By contrast, this design could also be limited in this age group since the patients might not be able to precisely localize their pain.^21^ Furthermore, each subgroup included only two studies. Considering these challenges, additional studies are needed to confirm the results of pillar suture on pain relief.

In the present study, pillar suture showed a statistically significant reduction in primary and secondary postoperative bleeding (ORs = 0.47 and 0.14, respectively). The pillar suture covers the open tonsillar fossa and can improve hemostasis by compressing the dead space, which can be the source of bleeding. In particular, rapid wound healing after tonsillectomy with the pillar suture may have resulted in a significant reduction in secondary postoperative bleeding.^7^ In addition, children over a certain age have been reported to have an increased risk of postoperative bleeding due to NSAIDs for pain control.^22^, ^23^ If post-tonsillectomy pain was reduced in children, the amount of analgesics used would be reduced, and drug-induced bleeding could be prevented. These observations could partially explain our favorable outcome regarding postoperative bleeding.

In our study, pillar edema was significantly increased due to pillar suture (OR = 9.55). Edema is caused by increased surgical manipulation of the mucosa and the mucosal tension from the suturing. Although pillar edema increased, the incidence of palatal discomfort sensation or suture site infection did not significantly increase, suggesting that the edema did not cause additional complications. Also, since the postoperative pain did not increase from the pillar suture, it implies that pain caused by the edema would not occur.

Velopharyngeal insufficiency may occur when the upper airway is structurally modified by excessive manipulation, which affects the position and height of the palate, including the uvula.^24^ Genc et al. had shown a higher incidence of velopharyngeal insufficiency in the pillar suture group than in the control group.^7^ However, our summated results showed no statistical significance in velopharyngeal insufficiency. The incidences of velopharyngeal insufficiency due to pillar suture in the included studies were rare and tonsillectomy alone could cause velopharyngeal insufficiency regardless of pillar suture.^25^ Therefore, it is difficult to determine the effect of pillar suture on velopharyngeal function.

The effect of pillar suture in tonsillectomy may be not only related to postoperative complications. Several studies have reported that pillar suturing with tonsillectomy is more effective than tonsillectomy alone in pediatric obstructive sleep apnea.^26^, ^27^ As a treatment for pediatric obstructive sleep apnea, the success rate of adenotonsillectomy has been reported to vary from 59.8% to 82.9%.^28^, ^29^, ^30^ There are several other treatment options besides adenotonsillectomy in pediatric obstructive sleep apnea.^31^ However, a simple additional pillar suturing with tonsillectomy could effectively extend the airway in pediatric patients, which may be another advantage of this technique. If further research is carried out in the future, the effect of pillar suture on respiratory symptoms such as snoring and sleep apnea will also be systematically assessed.

This study had several limitations. The number of studies was small because only randomized controlled trials were included. We did not differentiate between tonsillectomy methods, either cold dissection or cautery dissection, and all were included in the analysis. Most studies examined the effect of pillar suture on subjects by performing the same tonsillectomy method throughout, so meta-analysis was reasonable. In addition, there were differences in pillar suture types for each study. Catgut was the suture primarily used, but there were also studies that used polydioxanone or polyglactin 910. This could be a confounding factor in measuring the effect of pillar suture.

## Conclusions

This meta-analysis demonstrates that pillar suture in conjunction with pediatric tonsillectomy increases postoperative edema, but reduces the incidence of primary and secondary postoperative bleeding. Postoperative pain was significantly reduced only in the subgroups comparing the effect of pillar suture in a single patient. However, because of the low number of included studies in each group, further studies are needed to confirm the pain-relieving effect of pillar suture. Palatal hematoma, palatal discomfort sensation, site infection, and velopharyngeal insufficiency showed no significant differences between the pillar suture and control groups.

## Conflicts of interest

The authors declare no conflicts of interest.

## References

[1] Ayatollahi V., Behdad S., Hatami M., Moshtaghiun H., Baghianimoghadam B. (2012). Comparison of peritonsillar infiltration effects of ketamine and tramadol on post tonsillectomy pain: a double-blinded randomized placebo-controlled clinical trial. Croat Med J..

[2] Vaiman M., Eviatar E., Shlamkovich N., Segal S. (2003). Effect of modern fibrin glue on bleeding after tonsillectomy and adenoidectomy. Ann Otol Rhinol Laryngol..

[3] Stoeckli S.J., Moe K.S., Huber A., Schmid S. (1999). A prospective randomized double-blind trial of fibrin glue for pain and bleeding after tonsillectomy. Laryngoscope..

[4] Akural E.I., Alahuhta S., Ohtonen P., Lopponen H. (2016). Peritonsillar morphine infiltration to prevent early postoperative pain after tonsillectomy: A randomised controlled trial. Eur J Anaesthesiol..

[5] Nandapalan V., McIlwain J.C. (1995). Tonsillar fossa obliteration and post-operative pain. Clin Otolaryngol Allied Sci..

[6] Kerekhanjanarong V., Tang-On N., Supiyaphun P., Sastarasadhit V. (2001). Tonsillar fossa steroid injection for reduction of the post-tonsillectomy pain. J Med Assoc Thai..

[7] Genc E., Hanci D., Ergin N.T., Dal T. (2006). Can mucosal sealing reduce tonsillectomy pain?. Int J Pediatr Otorhinolaryngol..

[8] Elkholy T.A. (2016). Modified surgical technique with pillars repair in reducing post tonsillectomy haemorrhage. Int Inv J Med Med Sci..

[9] Akcan F.A., Dundar Y. (2018). Posterior pillar mucosal suspension technique for posttonsillectomy pain and wound healing: a prospective, randomized, controlled trial. Eur Arch Otorhinolaryngol..

[10] Sakallioglu O., Duzer S., Kapusuz Z. (2014). The efficiacy of anterior and posterior archs suturation at inferior tonsillar pole for posttonsillectomy pain control. Indian J Otolaryngol Head Neck Surg..

[11] Ramjettan S., Singh B. (1996). Are sutured faucial pillars really an advantage in tonsillectomy?. S Afr J Surg..

[12] Matt B.H., Krol B.J., Ding Y., Juliar B.E. (2012). Effect of tonsillar fossa closure on postoperative pain and bleeding risk after tonsillectomy. Int J Pediatr Otorhinolaryngol..

[13] Liu J.H., Anderson K.E., Willging J.P., Myer C.M., Shott S.R., Bratcher G.O. (2001). Posttonsillectomy hemorrhage: what is it and what should be recorded?. Arch Otolaryngol Head Neck Surg..

[14] Cohen N., Sommer D.D. (2016). Post-tonsillectomy pain control: consensus or controversy?. Pain Manag..

[15] Chapman C.R., Tuckett R.P., Song C.W. (2008). Pain and stress in a systems perspective: reciprocal neural, endocrine, and immune interactions. J Pain..

[16] Gross C.W., Gallagher R., Schlosser R.J., Burks S.G., Flanagan H.L., Mintz P.D. (2001). Autologous fibrin sealant reduces pain after tonsillectomy. Laryngoscope..

[17] Sclafani A.P., Jacono A.A., Dolitsky J.N. (2001). Grafting of the peritonsillar fossa with an acellular dermal graft to reduce posttonsillectomy pain. Am J Otolaryngol..

[18] Isaacson G. (2012). Tonsillectomy care for the pediatrician. Pediatrics..

[19] Lavy J.A. (1997). Post-tonsillectomy pain: the difference between younger and older patients. Int J Pediatr Otorhinolaryngol..

[20] Sendi K.S., Zawawi F.T., Al-Amry S., Alnoury I., Al-Radadi A.M. (2009). Tonsillar fossa closure: a quest in tonsillectomy pain reduction. JKAU: Med Sci..

[21] Fornazieri M.A., Miyazato E.S., Yamamoto H.M., de Lima Navarro P., de Rezende Pinna F., Voegels R.L. (2018). Reducing the exposure of the tonsillar fossa does not impact postoperative pain levels in children undergoing tonsillectomy: a double-blind randomized controlled trial. Int J Pediatr Otorhinolaryngol..

[22] Swanson R.T., Schubart J.R., Carr M.M. (2018). Association of ibuprofen use with post-tonsillectomy bleeding in older children. Am J Otolaryngol..

[23] Smith I., Wilde A. (1999). Secondary tonsillectomy haemorrhage and non-steroidal anti-inflammatory drugs. J Laryngol Otol..

[24] Shu Y., Yao H.B., Yang D.Z., Wang B. (2018). Postoperative characteristics of combined pharyngoplasty and tonsillectomy versus tonsillectomy in children with obstructive sleep apnea syndrome. Arch Argent Pediatr..

[25] Hu T.L., Yun C., Wang R., Chen P.K., Lo L.J. (2008). Management of velopharyngeal insufficiency in the presence of enlarged tonsils: comparing a one-stage versus two-stage treatment result. J Plast Reconstr Aesthet Surg..

[26] Friedman M., Samuelson C.G., Hamilton C., Maley A., Taylor D., Kelley K. (2012). Modified adenotonsillectomy to improve cure rates for pediatric obstructive sleep apnea: a randomized controlled trial. Otolaryngol Head Neck Surg..

[27] Chiu P.H., Ramar K., Chen K.C., Tsai Y.J., Lin C.M., Chiang Y.C. (2013). Can pillar suturing promote efficacy of adenotonsillectomy for pediatric OSAS? A prospective randomized controlled trial. Laryngoscope..

[28] Friedman M., Wilson M., Lin H.C., Chang H.W. (2009). Updated systematic review of tonsillectomy and adenoidectomy for treatment of pediatric obstructive sleep apnea/hypopnea syndrome. Otolaryngol Head Neck Surg..

[29] Marcus C.L., Moore R.H., Rosen C.L., Giordani B., Garetz S.L., Taylor H.G. (2013). A randomized trial of adenotonsillectomy for childhood sleep apnea. N Engl J Med..

[30] Brietzke S.E., Gallagher D. (2006). The effectiveness of tonsillectomy and adenoidectomy in the treatment of pediatric obstructive sleep apnea/hypopnea syndrome: a meta-analysis. Otolaryngol Head Neck Surg..

[31] Praud J.P., Dorion D. (2008). Obstructive sleep disordered breathing in children; beyond adenotonsillectomy. Pediatr Pulmonol..

